# RDW-to-Albumin Ratio as a Simple Biomarker for Early Mortality Risk After LVAD Implantation

**DOI:** 10.3390/medicina62050853

**Published:** 2026-04-30

**Authors:** İbrahim Demir, Bilge Ecemiş, Ayşe Zorba, Selinsu Güleşce, Yahya Yıldız, İbrahim Oğuz Karaca, Korhan Erkanlı

**Affiliations:** 1 Department of Cardiovascular Surgery, Istanbul Medipol University, Istanbul 34214, Turkey; bilge.yilmaz@medipol.edu.tr (B.E.); ayse.zorba@medipol.edu.tr (A.Z.); kerkanli@medipol.edu.tr (K.E.); 2 Faculty of Medicine, Istanbul Medipol University, Istanbul 34214, Turkey; selinsu.gulesce@std.medipol.edu.tr; 3 Department of Anesthesiology and Reanimation, Istanbul Medipol University, Istanbul 34214, Turkey; yahya.yildiz@medipol.edu.tr; 4 Department of Cardiology, Istanbul Medipol University, Istanbul 34214, Turkey; iokaraca@medipol.edu.tr

**Keywords:** left ventricular assist device, in-hospital mortality, RDW, albumin, INTERMACS

## Abstract

*Background and Objectives:* Early risk stratification remains challenging in patients undergoing left ventricular assist device (LVAD) implantation. Red cell distribution width (RDW) and serum albumin reflect systemic stress and nutritional reserve; their ratio (RDW-to-albumin ratio, RAR) may provide a simple preoperative index. We evaluated whether preoperative RAR is associated with early mortality after LVAD implantation. *Materials and Methods:* We conducted a retrospective cohort study of LVAD recipients (2019–2025). RAR was calculated as RDW (%) divided by albumin (g/dL) from preoperative blood tests obtained 24–48 h before surgery. The primary endpoint was in-hospital mortality. The secondary endpoint was 90-day survival. In-hospital mortality was analyzed using logistic regression with parsimonious adjustment for INTERMACS high-risk status (profiles 1–2 vs. 3–7); penalized regression was used to reduce small-sample bias. Discrimination was assessed using receiver operating characteristic (ROC) analysis. Ninety-day survival was evaluated using Cox proportional hazards models. *Results:* Forty-seven patients were included (37 survivors; 10 in-hospital deaths). Higher RAR was associated with increased odds of in-hospital mortality and remained significant after adjustment for INTERMACS high-risk status (OR 1.68, 95% CI 1.04–2.90). INTERMACS high-risk status was strongly associated with in-hospital mortality (OR 17.89, 95% CI 3.19–138.07). RAR demonstrated good discrimination for in-hospital mortality (AUC 0.801, 95% CI 0.648–0.955). For 90-day survival, RAR showed a borderline association in unadjusted analysis (HR 1.28, 95% CI 0.98–1.68) and was not significant after adjustment (HR 1.20, 95% CI 0.89–1.63). *Conclusions:* In this small single-center cohort, preoperative RAR was independently associated with in-hospital mortality after LVAD implantation. These findings should be considered hypothesis-generating and require external validation.

## 1. Introduction

Durable left ventricular assist device (LVAD) therapy has become an established option for patients with end-stage heart failure, yet early postoperative outcomes remain heterogeneous. This creates a practical need for simple, objective, and widely available pre-implant markers that can help identify patients at higher risk for early mortality and guide perioperative planning. Traditional risk stratification frameworks—such as clinical acuity profiles (e.g., INTERMACS)—capture hemodynamic urgency but do not fully reflect the biological “reserve” that often determines early postoperative resilience [1,2].

Red cell distribution width (RDW) and serum albumin are routinely measured biomarkers that have been associated with adverse outcomes in cardiovascular disease. RDW likely reflects a composite of systemic stress processes, whereas albumin captures nutritional and inflammatory reserve. Combining these measures into a single index—the RDW-to-albumin ratio (RAR)—may provide a pragmatic summary of perioperative vulnerability using laboratory data already obtained as part of standard preoperative assessment [3,4,5].

However, evidence on RAR in the LVAD setting is limited. In this study, we evaluated the association of preoperative RAR with in-hospital mortality (primary endpoint) and 90-day survival (secondary endpoint) after LVAD implantation, with parsimonious adjustment for clinical severity. We additionally explored associations between RAR and fibrosis-/score-based measures as hypothesis-generating analyses [2,6].

## 2. Materials and Methods

### 2.1. Study Design and Population

This retrospective observational cohort study included consecutive adult patients who underwent durable left ventricular assist device (LVAD) (HeartMate; Abbott: Chicago, IL, USA) implantation at our institution between 2019 and 2025. Clinical, laboratory, echocardiographic, and invasive hemodynamic data were retrieved from institutional medical records using a predefined data extraction form. Patients were eligible if they had complete ascertainment of the primary endpoint (in-hospital mortality) and preoperative laboratory measurements required to calculate the RDW-to-albumin ratio (RAR). We designed the study to evaluate a pragmatic, routinely available biomarker for early perioperative risk stratification in a real-world LVAD population [3].

Preoperative blood tests used for biomarker calculations were obtained as part of routine preoperative assessment 24–48 h before LVAD implantation. This window was chosen to reflect the patient’s immediate preoperative physiological state and to maintain consistency across patients while minimizing postoperative confounding [1].

The primary endpoint was in-hospital mortality, defined as death occurring during the index hospitalization for LVAD implantation. The secondary endpoint was 90-day mortality, derived from follow-up time and death status, with follow-up administratively truncated at 90 days to focus on early post-implant outcomes [2].

### 2.2. Main Predictor: RDW-to-Albumin Ratio

The main exposure of interest was the RDW-to-albumin ratio (RAR), calculated as RAR = RDW (%)/albumin (g/dL), using values from this preoperative sampling window. RAR was analyzed as a continuous variable (per 1-unit increase). For descriptive visualization of risk gradients, RAR was additionally summarized by quartiles [4].

### 2.3. Covariates and Baseline Variables

Baseline variables included age, sex, BMI, NYHA class, comorbidities, routine laboratory values, and preoperative echocardiographic/hemodynamic measurements as available. Given the limited number of primary outcome events, multivariable adjustment was restricted a priori to a parsimonious clinical severity covariate: INTERMACS high-risk status defined as profiles 1–2 vs. 3–7 [7].

### 2.4. Statistical Analysis

Continuous variables were summarized as median (Q1, Q3) and compared using the Wilcoxon rank-sum test. Categorical variables were summarized as *n* (%) and compared using Fisher’s exact test (selected due to small cell counts). Two-sided *p*-values < 0.05 were considered statistically significant.

### 2.5. Primary Analysis: In-Hospital Mortality

We evaluated the association between RAR and in-hospital mortality using the following:

1. Unadjusted logistic regression;
2. Firth’s penalized logistic regression to mitigate small-sample bias and separation risk;
3. Adjusted models including RAR + INTERMACS as a parsimonious confounder set.

Effect sizes were reported as odds ratios (ORs) with 95% confidence intervals (CIs). Discrimination was assessed using ROC curves and AUC with confidence intervals. Risk gradients were visualized by mortality proportions across RAR quartiles [8].

### 2.6. Secondary Analysis: 90-Day Survival

For 90-day outcomes, time-to-event analyses were performed using Cox proportional hazards models, including unadjusted and INTERMACS-adjusted models. The proportional hazards assumption was checked using Schoenfeld residuals. Kaplan–Meier curves were generated as supportive visualization using a median split of RAR, with log-rank testing [9].

All analyses were performed using R version 4.5.2 (R Foundation for Statistical Computing, Vienna, Austria).

### 2.7. Exploratory Analyses

Exploratory analyses were hypothesis-generating. The association between RAR and fibrosis rate was assessed using Spearman correlation and visualized with scatterplots and nonparametric smoothing. Discrimination of high fibrosis by RAR was evaluated using ROC/AUC (DeLong 95% CI) and Firth penalized logistic regression (unadjusted and adjusted for INTERMACS high-risk). Differences in RAR between high THS groups were assessed using the Wilcoxon rank-sum test, and the association between RAR and THS-10 score was evaluated using Spearman correlation. All exploratory analyses used complete-case data for the variables included, and the corresponding sample size is reported with results.

### 2.8. Missing Data

Analyses were conducted using complete-case data for each model (i.e., participants were included if they had non-missing values for the outcome and covariates used in that analysis). Denominators therefore vary across variables and analyses. No imputation was performed.

## 3. Results

A total of 47 LVAD recipients were included in the primary (in-hospital) outcome analyses. In-hospital mortality occurred in 10 patients (21.3%), while 37 patients (78.7%) survived to discharge. Baseline characteristics are summarized in Table 1. Age and BMI were not significantly different between groups (median age 60 vs. 69 years, *p* = 0.093; BMI 28.7 vs. 30.1 kg/m^2^, *p* = 0.90). The distribution of sex and NYHA class did not differ significantly (*p* = 0.70 and *p* > 0.90, respectively). To improve readability, a condensed set of clinically relevant baseline variables is presented in Table 1, while the full baseline dataset is provided in Supplementary Table S1.

Clinical severity at implantation differed by INTERMACS profile (*p* = 0.003). Non-survivors were enriched for INTERMACS profiles 1–2, consistent with higher acuity at implantation.

Among laboratory biomarkers, albumin was lower in non-survivors (3.23 vs. 3.71 g/dL, *p* = 0.015). RDW alone showed a trend toward higher values in non-survivors (16.35 vs. 15.0, *p* = 0.073). The composite RDW/Albumin ratio (RAR) was markedly higher in non-survivors (5.28 vs. 3.97, *p* = 0.004). Other laboratory and hemodynamic variables did not show statistically significant differences at baseline, although PAPi tended to be lower in non-survivors (2.40 vs. 3.11, *p* = 0.11).

### 3.1. Association of RAR with In-Hospital Mortality

**Figure 1:**
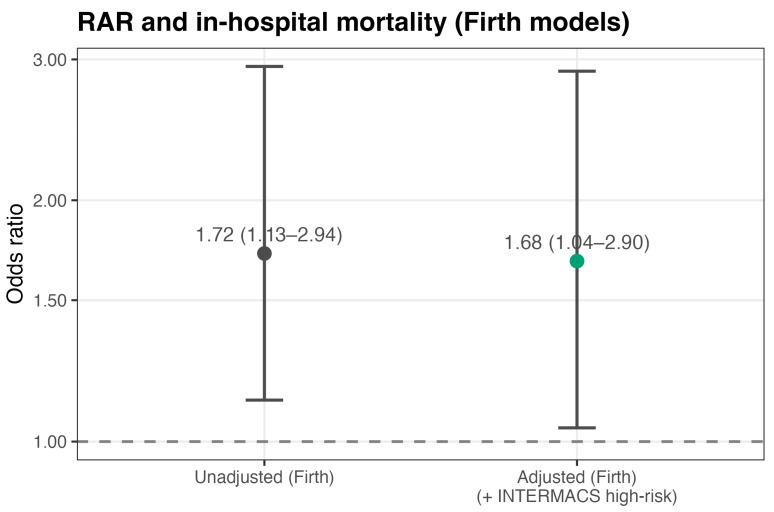
Association between preoperative RAR and in-hospital mortality (Firth models). Forest plot showing odds ratios (ORs) with 95% confidence intervals for in-hospital mortality per 1-unit increase in RAR from unadjusted and INTERMACS-adjusted Firth penalized logistic regression models (INTERMACS 1–2 vs. 3–7).

Primary association models are presented in Table 2, with effect estimates expressed per 1-unit increase in RAR. In unadjusted logistic regression, higher RAR was associated with increased odds of in-hospital mortality (OR **1.82**, *p* = 0.019). Given the limited number of events, Firth penalized logistic regression was also applied and yielded a consistent association (OR **1.72**, 95% CI 1.13–2.94, *p* = 0.012) (Figure 1).

In parsimonious adjusted models including INTERMACS high-risk status (profiles 1–2 vs. 3–7), RAR remained independently associated with in-hospital mortality in both standard logistic regression (OR **1.78**, *p* = 0.038) and the Firth model (OR **1.68**, 95% CI 1.04–2.90, *p* = 0.035). INTERMACS high-risk status was strongly associated with in-hospital mortality (adjusted Firth OR **17.89**, 95% CI 3.19–138.07, *p* < 0.001).

### 3.2. Discrimination and Risk Gradient of RAR for In-Hospital Mortality

**Figure 2:**
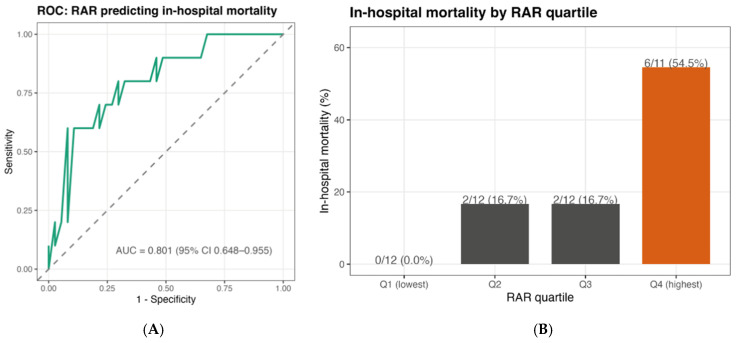
Discrimination and risk gradient of RAR for in-hospital mortality. (**A**) ROC curve for preoperative RAR predicting in-hospital mortality with AUC (95% CI). (**B**) In-hospital mortality across RAR quartiles (Q1–Q4) with event counts (n/N, %).

RAR demonstrated good discriminatory performance for in-hospital mortality, with an AUC of **0.801** (95% CI 0.648–0.955). Using the Youden index, the optimal internal threshold for RAR was 5.21, yielding a sensitivity of 60% and specificity of 91.9%. At this operating point, PPV and NPV were 66.7% and 89.5%, respectively (PPV/NPV are prevalence-dependent), and the threshold should be considered exploratory pending external validation. When stratified by quartiles, in-hospital mortality was concentrated in the highest RAR quartile (Q4), whereas no deaths occurred in Q1 (Figure 2A,B).

In a complete-case subset with available INTERMACS data (*n* = 45), AUCs were 0.798 for RAR alone, 0.806 for INTERMACS high-risk alone, and 0.901 for the combined RAR+INTERMACS model. DeLong tests showed no difference between RAR and INTERMACS (*p* = 0.946), while the combined model showed higher AUC without reaching conventional significance versus RAR alone (*p* = 0.162) and with a trend versus INTERMACS alone (*p* = 0.070).

### 3.3. 90-Day Survival

**Figure 3:**
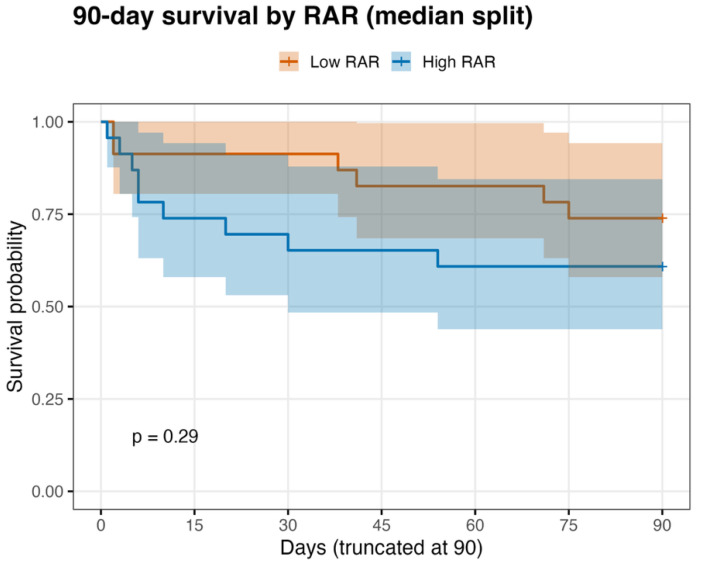
Ninety-day survival by preoperative RAR (median split). Kaplan–Meier curves up to 90 days after LVAD implantation stratified by low vs. high RAR defined by the cohort median. Shaded areas indicate 95% confidence intervals; *p*-value from the log-rank test.

Ninety-day survival analyses were performed using time-to-event data, with 15 deaths occurring within 90 days (Figure 3). In unadjusted Cox regression, RAR showed a borderline association with 90-day mortality (HR **1.28**, 95% CI 0.98–1.68, *p* = 0.069). After adjustment for INTERMACS high-risk status, the association between RAR and 90-day mortality was attenuated and not statistically significant (adjusted HR **1.20**, 95% CI 0.89–1.63, *p* = 0.229), whereas INTERMACS high-risk status remained significantly associated with 90-day mortality (HR **5.29**, 95% CI **1**.79–15.63, *p* = 0.003) (Table 3).

### 3.4. Exploratory Analyses (Supportive)

Exploratory analyses (*n* = 36) are summarized briefly here and presented in full in the Supplementary Materials (Figures S1–S4). RAR showed a weak positive correlation with fibrosis rate (Spearman ρ = 0.27, *p* = 0.113) and modest discrimination for high fibrosis (AUC = 0.683, 95% CI 0.494–0.873). RAR correlated positively with THS-10 score (Spearman ρ = 0.34, *p* = 0.043) and showed a trend toward higher values in high THS score vs. low THS score groups (*p* = 0.064). These findings were considered hypothesis-generating and were not used to redefine the primary endpoint conclusions.

## 4. Discussion

In this single-center LVAD cohort, preoperative RDW/Albumin ratio (RAR), measured 24–48 h before implantation, was associated with in-hospital mortality and showed good discrimination (AUC ~0.80), including in parsimoniously adjusted penalized (Firth) models incorporating INTERMACS high-risk status [3]. Given the small sample size and limited number of events, effect estimates are imprecise and should be interpreted as hypothesis-generating pending external validation.

RAR combines two routinely available measures that reflect complementary dimensions of perioperative vulnerability (systemic stress biology and nutritional/inflammatory reserve). This integrated signal may explain why RAR showed clearer separation by in-hospital outcome than RDW alone in our cohort, consistent with reports linking RDW- and albumin-related markers to vulnerability in other cardiovascular settings [10,11,12].

In-hospital mortality is temporally closest to the preoperative physiological state captured by RAR. The attenuation of the RAR association at 90 days after adjusting for INTERMACS suggests that RAR may function primarily as a perioperative risk marker, while later outcomes may be more strongly influenced by overall clinical severity and post-implant course [2,7]. To delineate levels of evidence, our primary analysis (RAR and in-hospital mortality) is the confirmatory component of this study, whereas 90-day survival results are supportive and exploratory given limited power. Analyses linking RAR with fibrosis and THS-derived measures are hypothesis-generating and should not be interpreted mechanistically.

INTERMACS high-risk status remained a dominant predictor of early outcomes, as expected. In this dataset, RAR showed an independent association with in-hospital mortality after parsimonious adjustment, suggesting that it may capture complementary biological information; however, given the limited sample size, the incremental value of RAR beyond established risk stratification requires confirmation in larger cohorts [13,14,15].

Exploratory analyses relating RAR to fibrosis burden and THS-derived measures were limited by missingness and sample size. In this cohort, RAR showed more consistent association with THS-10 than with fibrosis metrics; however, these findings are hypothesis-generating and should not be interpreted mechanistically [8,16].

### 4.1. Strengths

A strength of this study is the evaluation of an inexpensive biomarker derived from routinely collected preoperative laboratory tests in a consistent 24–48 h window. We also used penalized regression to reduce small-sample bias in sparse-event modeling.

### 4.2. Limitations

This study has important limitations. First, the cohort is small and single-center, which limits generalizability and increases the risk of imprecise estimates; confidence intervals are therefore wide and subgroup inferences are not justified. Second, the number of events is limited; although we used penalized regression to reduce small-sample bias, the findings should still be interpreted cautiously and require external validation. We could not benchmark RAR against HMRS/DTRS because required variables (e.g., INR) were not available in our dataset. Third, missing data limited additional adjustment and constrained interpretation of exploratory analyses. Fourth, RAR is a composite index and may be influenced by unmeasured perioperative factors (e.g., occult infection/inflammation, hepatic congestion, transfusion burden), which could contribute to residual confounding. Finally, the attenuation of the RAR association at 90 days suggests that RAR should not be positioned as a stand-alone short-term survival predictor; rather, it may be most relevant to the immediate perioperative window [5].

### 4.3. Future Directions

Future work should validate RAR in larger multi-center LVAD cohorts, test calibration (not just discrimination), and evaluate whether adding RAR to existing clinical models meaningfully improves risk classification. It will also be important to explore whether RAR is modifiable (e.g., through nutritional and inflammatory optimization) and whether changes in RAR between evaluation and implantation carry incremental prognostic information [17,18].

## 5. Conclusions

Preoperative RAR was associated with in-hospital mortality after LVAD implantation and showed good discrimination in this single-center cohort. Given limited precision, these findings should be considered hypothesis-generating and warrant external validation before clinical use.

## Figures and Tables

**Table 1:** Baseline characteristics stratified by in-hospital mortality.

Variable	Survivor (*n* = 37)	Non-Survivor (*n* = 10)	*p*-Value
**Demographics**			
Age	60 (53–71)	69 (63–70)	0.093
BMI (kg/m^2^)	28.7 (24.3–33.2)	30.1 (25.6–32.0)	0.9
Sex			0.7
Male	30 (81%)	9 (90%)	
Female	7 (19%)	1 (10%)	
NYHA			>0.9
1	0 (0%)	0 (0%)	
2	1 (2.7%)	0 (0%)	
3	3 (8.1%)	0 (0%)	
4	33 (89%)	10 (100%)	
INTERMACS Profile			***0.003***
1	1 (2.8%)	3 (33%)	
2	1 (2.8%)	3 (33%)	
3	11 (31%)	0 (0%)	
4	15 (42%)	3 (33%)	
5	5 (14%)	0 (0%)	
6	2 (5.6%)	0 (0%)	
7	1 (2.8%)	0 (0%)	
**Blood Tests**			
Creatinine (mg/dL)	1.30 (1.01–1.82)	1.66 (1.13–2.67)	0.3
Albumin (g/dL)	3.71 (3.33–4.24)	3.23 (2.73–3.59)	***0.015***
RDW (%)	15 (13.7–16.1)	16.35 (14.8–17.5)	0.073
CRP (mg/L)	19 (9–53)	51 (11–80)	0.3
NT-proBNP (pg/mL)	5107 (3476–9847)	7062 (5946–8115)	0.5
RDW/Albumin Ratio	3.97 (3.49–4.75)	5.28 (4.64–5.83)	***0.004***
**Cardiac Measurements**			
PCWP (mmHg)	25 (16–30)	30 (30–35)	0.2
PAPi	3.11 (2.31–4.00)	2.40 (1.45–3.39)	0.11
LVEF (%)			0.2
10	1 (2.8%)	1 (10%)	
15	8 (22%)	0 (0%)	
20	23 (64%)	7 (70%)	
25	4 (11%)	2 (20%)	
**Comorbidities**			
Hypertension	28 (80%)	8 (89%)	>0.9
Diabetes Mellitus	19 (51%)	5 (50%)	>0.9
Chronic Kidney Disease	18 (49%)	5 (50%)	>0.9
Coronary Artery Disease	27 (73%)	8 (80%)	>0.9

**Table 2:** Primary analysis: Association of RAR with in-hospital mortality.

Model	Predictor	OR (95% CI)	*p*-Value
Unadjusted (Generalized Linear Model)	RDW/Albumin Ratio	1.82 (1.10–3.02)	0.019
Unadjusted (Firth’s Penalized Logistic Regression)	RDW/Albumin Ratio	1.72 (1.13–2.94)	0.012
Adjusted (Generalized Linear Model)	RDW/Albumin Ratio	1.78 (1.03–3.08)	0.038
Adjusted (Firth’s Penalized Logistic Regression)	RDW/Albumin Ratio	1.68 (1.04–2.90)	0.035
Adjusted (Generalized Linear Model)	INTERMACS (1–2 vs. 3–7)	24.40 (3.25–183.18)	0.002
Adjusted (Firth’s Penalized Logistic Regression)	INTERMACS (1–2 vs. 3–7)	17.89 (3.19–138.07)	<0.001

**Table 3:** Secondary endpoint (90-day survival): Association of RAR with mortality (Cox proportional hazards models).

Model	Predictor	HR (95% CI)	*p*-Value
Unadjusted Cox	RAR (per 1-unit)	1.28 (0.98–1.68)	0.069
Adjusted Cox	RAR (per 1-unit)	1.20 (0.89–1.63)	0.229
Adjusted Cox	INTERMACS (1–2 vs. 3–7)	5.29 (1.79–15.63)	0.003

## Data Availability

De-identified individual participant data are not publicly available due to institutional and regulatory restrictions. Additional information supporting the findings of this study can be made available from the corresponding author upon reasonable request, contingent upon institutional approval and data-sharing agreements.

## Supplementary Materials

The following supporting information can be downloaded at: https://www.mdpi.com/article/10.3390/medicina62050853/s1, Figure S1: Association between RDW/albumin ratio and myocardial fibrosis burden, Figure S2: Receiver operating characteristic analysis of RDW/albumin ratio for predicting high myocardial fibrosis burden, Figure S3: RDW/albumin ratio according to THS-high status, Figure S4: Association between RDW/albumin ratio and THS-10 score, Table S1: Expanded baseline characteristics stratified by in-hospital mortality.
