# Supplementary material for: RDW-to-Albumin Ratio as a Simple Biomarker for Early Mortality Risk After LVAD Implantation
Source: Medicina (Kaunas). 2026 Apr 30;62(5):853. doi: 10.3390/medicina62050853 (PMC13208264; doi:10.3390/medicina62050853)
Supplement: Supplementary file 1 [file medicina-62-00853-s001.zip › Table S1.pdf]

Table S1. Baseline characteristics stratified by in-hospital mortality.

Variable		Survivor (n=37)	Non-survivor (n=10)	*p-value*
**Demographics**				
Age		60 (53-71)	69 (63-70)	0.093
BMI (kg/m^2)		28.7 (24.3-33.2)	30.1 (25.6-32.0)	0.9
Sex				0.7
	Male	30 (81%)	9 (90%)	
	Female	7 (19%)	1 (10%)	
NYHA				>0.9
	1	0 (0%)	0 (0%)	
	2	1 (2.7%)	0 (0%)	
	3	3 (8.1%)	0 (0%)	
	4	33 (89%)	10 (100%)	
INTERMACS Profile				***0.003***
	1	1 (2.8%)	3 (33%)	
	2	1 (2.8%)	3 (33%)	
	3	11 (31%)	0 (0%)	
	4	15 (42%)	3 (33%)	
	5	5 (14%)	0 (0%)	
	6	2 (5.6%)	0 (0%)	
	7	1 (2.8%)	0 (0%)	
**Blood Tests**				
Haemoglobin (g/dL)		11.30 (9.70-12.90)	9.70 (8.30-12.10)	0.14
WBC (x10^9/L)		9.0 (6.8-10.8)	10.8 (6.5-12.9)	0.4
Platelets (x10^4/L)		193 (148-241)	215 (120-231)	0.7
Creatinine (mg/dL)		1.30 (1.01-1.82)	1.66 (1.13-2.67)	0.3
Albumin (g/dL)		3.71 (3.33-4.24)	3.23 (2.73-3.59)	***0.015***
RDW (%)		15 (13.7-16.1)	16.35 (14.8-17.5)	0.073
CRP (mg/L)		19 (9-53)	51 (11-80)	0.3
NT-proBNP (pg/mL)		5,107 (3,476-9,847)	7,062 (5,946-8,115)	0.5
RDW/Albumin Ratio		3.97 (3.49-4.75)	5.28 (4.64-5.83)	***0.004***
**Cardiac Measurements**				
TAPSE/PASP (mm/mmHg)		0.31 (0.24-0.40)	0.26 (0.25-0.34)	0.5
Cardiac Output (L/min)		3.75 (3.26-4.53)	4.10 (2.77-5.25)	0.5
Cardiac Index (L/min/m^2)		1.87 (1.68-2.10)	2.27 (1.52-2.49)	0.2
PCWP (mmHg)		25 (16-30)	30 (30-35)	0.2
PAPM (mmHg)		28 (22-40)	36 (20-40)	0.6
PAPi		3.11 (2.31-4.00)	2.40 (1.45-3.39)	0.11
LVEF (%)				0.2
	10	1 (2.8%)	1 (10%)	
	15	8 (22%)	0 (0%)	
	20	23 (64%)	7 (70%)	
	25	4 (11%)	2 (20%)	
PVR (Woods Unit)				>0.9
	1	14 (52%)	4 (67%)	
	2	2 (7.4%)	1 (17%)	
	3	4 (15%)	0 (0%)	
	4	3 (11%)	1 (17%)	
	5	1 (3.7%)	0 (0%)	
	6	2 (7.4%)	0 (0%)	

7	1 (3.7%)	0 (0%)	
**Comorbidities**			
Hypertension	28 (80%)	8 (89%)	>0.9
Diabetes Mellitus	19 (51%)	5 (50%)	>0.9
Chronic Kidney Disease	18 (49%)	5 (50%)	>0.9
Cerebrovascular Event	1 (2.7%)	1 (10%)	0.4
Atrial Fibrillation	12 (32%)	4 (40%)	0.7
Coronary Artery Disease	27 (73%)	8 (80%)	>0.9
Pacemaker Implantation	29 (78%)	8 (80%)	>0.9
