# Supplementary figures and images for: RDW-to-Albumin Ratio as a Simple Biomarker for Early Mortality Risk After LVAD Implantation
Source: Medicina (Kaunas). 2026 Apr 30;62(5):853. doi: 10.3390/medicina62050853 (PMC13208264; doi:10.3390/medicina62050853)

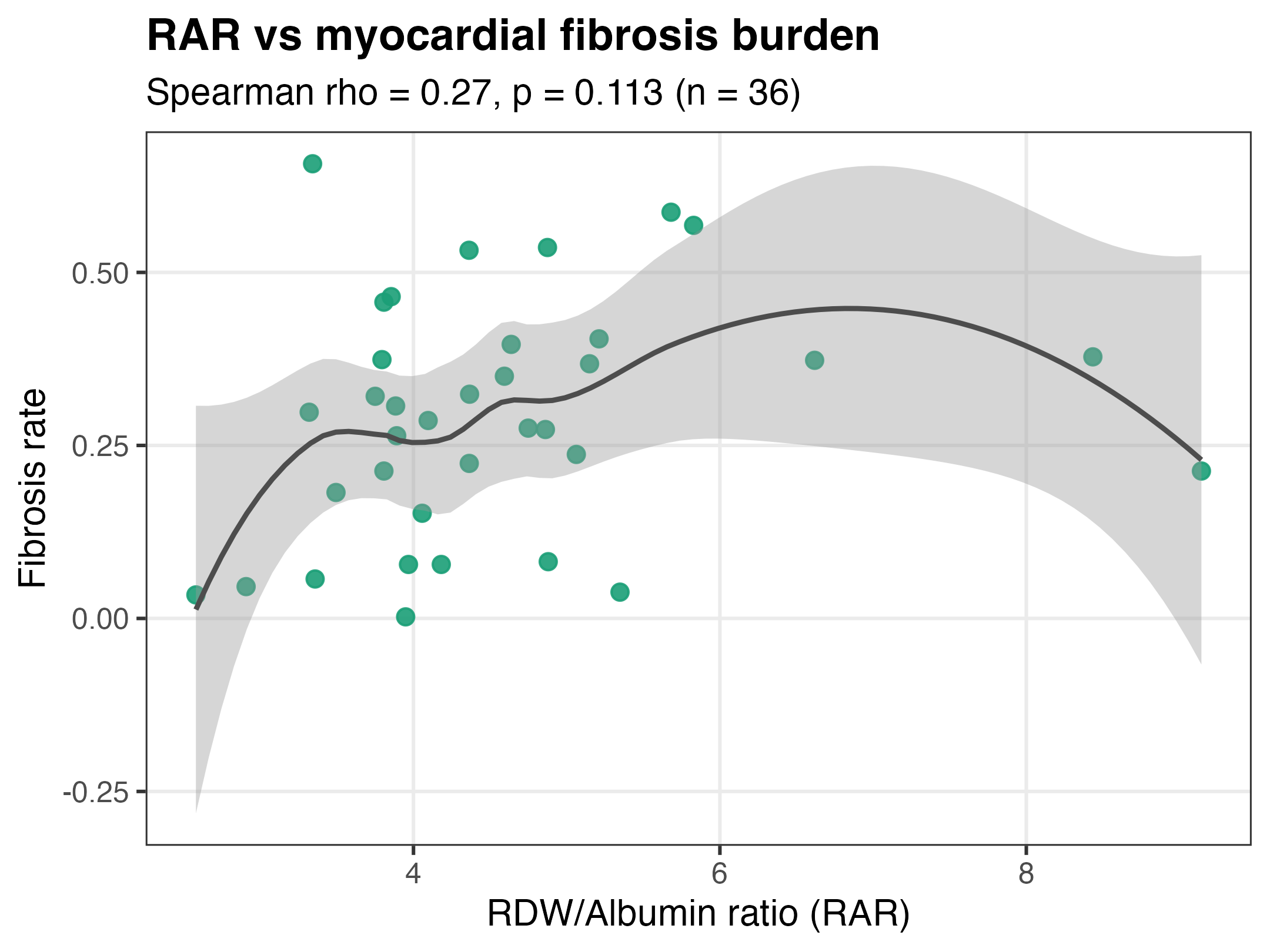

Supplement: Supplementary file 1 [file medicina-62-00853-s001.zip › S1.png]

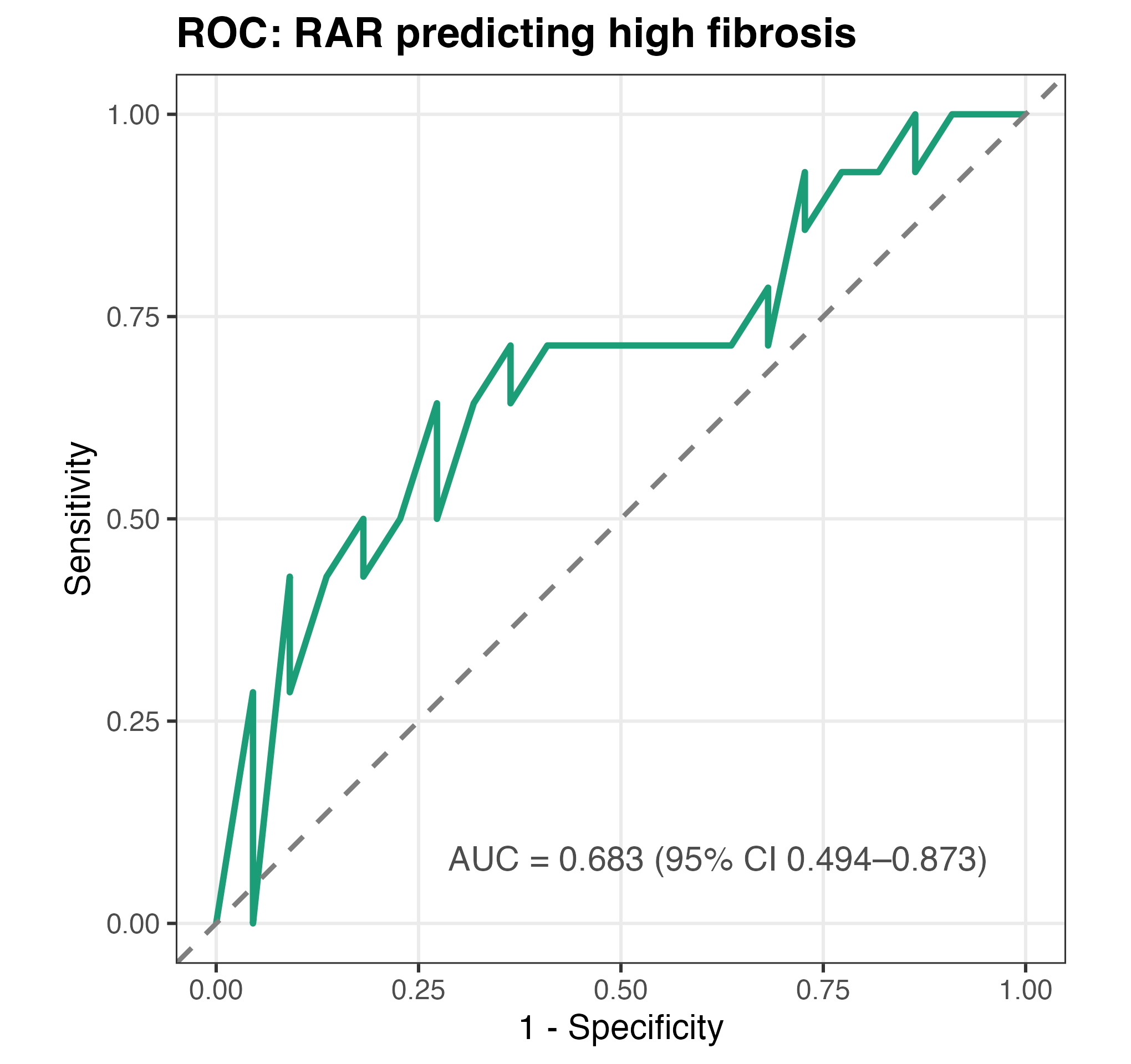

Supplement: Supplementary file 1 [file medicina-62-00853-s001.zip › S2.png]

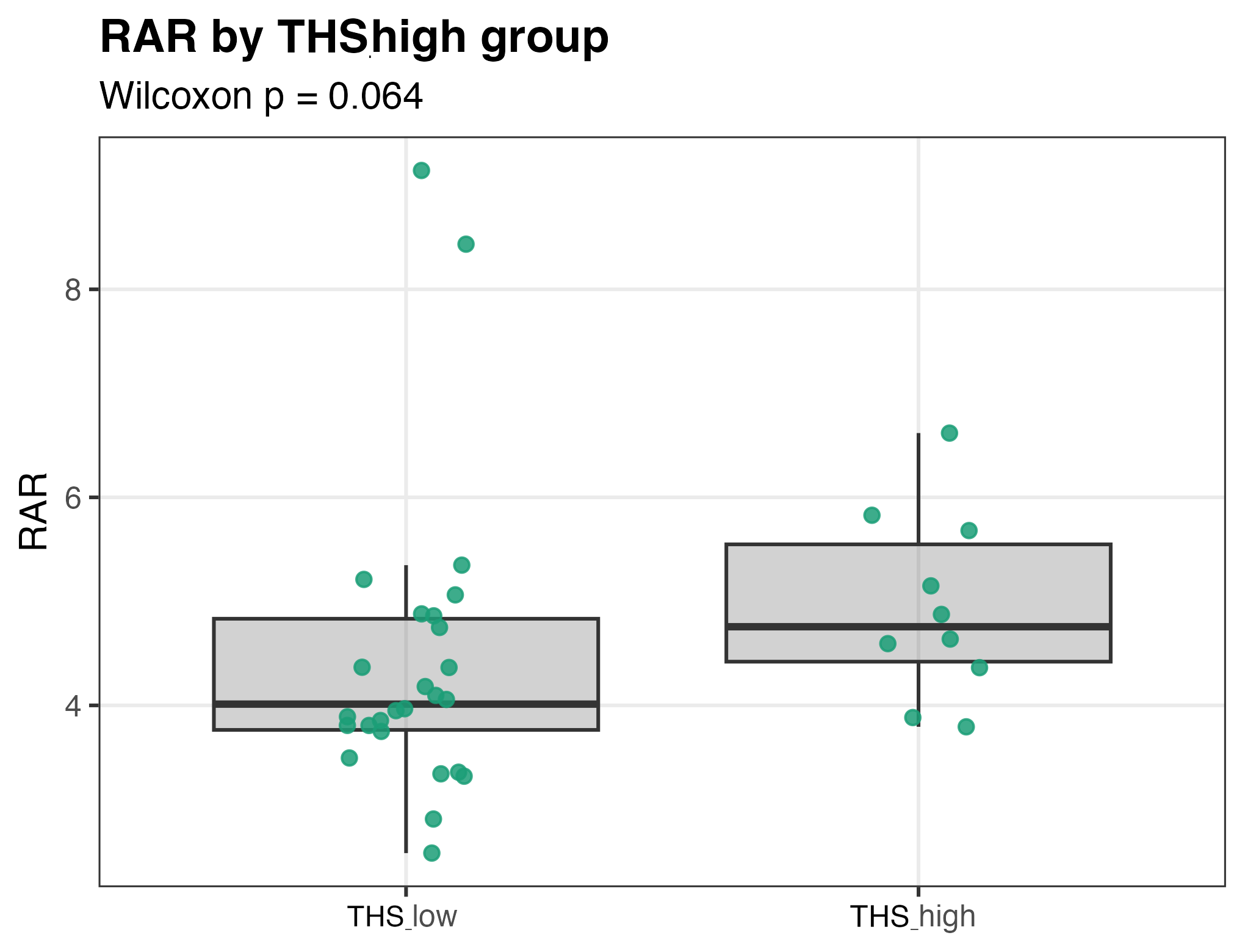

Supplement: Supplementary file 1 [file medicina-62-00853-s001.zip › S3.png]

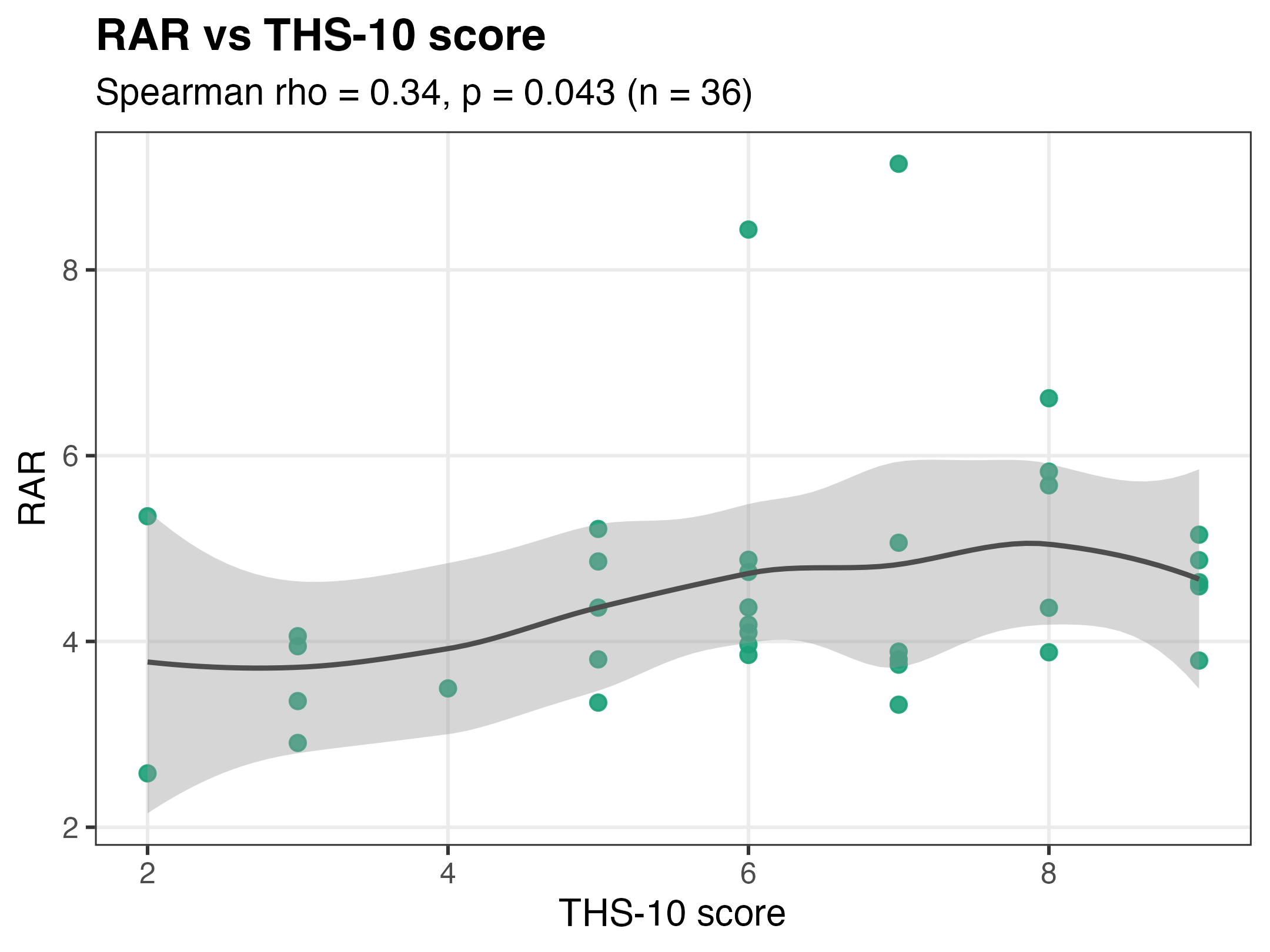

Supplement: Supplementary file 1 [file medicina-62-00853-s001.zip › S4.png]
